# Proposed Refinement of 2022 European LeukemiaNet Adverse-Risk Group of AML Patients Using a Real-World Cohort

**DOI:** 10.3390/cancers17091405

**Published:** 2025-04-23

**Authors:** Collins Wangulu, Davidson Zhao, Qianghua Zhou, Cuihong Wei, Rajat Kumar, Aaron Schimmer, Hong Chang

**Affiliations:** 1 Princess Margaret Cancer Biobank (PMCB), University Health Network, Toronto, ON M5G 2C4, Canada; collins.wangulu@uhn.ca; 2 Department of Laboratory Medicine and Pathobiology, University of Toronto, Toronto, ON M5S 1V4, Canada; 3 Department of Laboratory Hematology, University Health Network, Toronto, ON M5G 2C4, Canada; 4 Clinical Laboratory Genetics, Laboratory Medicine Program, University Health Network, Toronto, ON M5G 2C4, Canada; 5 Department of Hematology and Oncology, Princess Margaret Cancer Center, University Heath Network, Toronto, ON M5G 2C4, Canada

**Keywords:** 2022 European LeukemiaNet, adverse risk, *TP53* mutation, refinement, acute myeloid leukemia

## Abstract

**Background/Objectives:** The 2022 European LeukemiaNet (ELN 2022) is a widely used genotypic risk classification tool for the treatment and prognostication of acute myeloid leukemia (AML) patients. Our study evaluates its effectiveness in categorizing adverse-risk AML patients on standard therapy based on their overall survival (OS). **Methods**: We conducted a retrospective study involving 256 AML patients. **Results**: Those in the ELN 2022 adverse-risk group had the shortest OS (*p* < 0.0001) and were predominantly characterized by myelodysplasia-related (*MR*) mutations, complex karyotype (CK), monosomal karyotype (MK), and *TP53* mutation (*TP53 Mut*). Subclassification and analysis of this adverse-risk group based on the *TP53 Mut* status revealed a significantly shorter OS compared to the adverse *TP53* wild-type (*TP53 WT*) counterparts (*p* = 0.0036). We propose refining the ELN 2022 adverse-risk group into two categories, adverse *TP53 Mut* and adverse *TP53 WT* groups, to represent adverse- and ultra-adverse-risk groups, respectively. We used an external validation dataset to confirm our findings. **Conclusions**: This refinement allows for a more accurate classification of these adverse-risk patients based on their clinical outcomes.

## Simple Summary

The 2022 European LeukemiaNet (ELN 2022) risk classification is widely used for the treatment and prognostication of patients with acute myeloid leukemia (AML). The ELN 2022 adverse-risk category comprises a genotypically diverse patient population, which limits its effectiveness in accurately categorizing them based on their overall survival (OS). Recent validation studies have highlighted this, but with varied and conflicting findings. The studies were also constrained by their non-uniform patient selection, relatively younger cohorts, and low numbers of *TP53* mutant AML patients. Our study aims to demonstrate the heterogeneous nature of the ELN 2022 adverse-risk group in a real-world setting and to subclassify them to reflect their OS. Findings from our study will be used to cluster these patients into homogeneous and reproducible risk groups. Clinicians and researchers will be better equipped to make more precise predictions regarding expected clinical outcomes, enabling them to select the most appropriate treatment strategies.

## 1. Introduction

Acute myeloid leukemia (AML) is a hematopoietic malignancy characterized by a high genetic heterogeneity with distinct cytogenetic and genomic alterations that confer varied biological and clinical behavior within the disease spectrum [1]. Globally, the estimated age-standardized incidence rate of AML ranges from 0.70 to 3.23 cases per 100,000 persons and accounts for the majority of adult leukemia cases [2]. It has a median age at diagnosis of 68 years and an estimated 5-year overall survival (OS) of 30% [3]. Prognostic classification of AML based on genotypic subgroups is the core principle in the selection and individualization of treatment and is based on consensus guidelines established by the European LeukemiaNet in 2022 (ELN 2022) [4].

The ELN 2022 categorizes individuals with AML into favorable-, intermediate-, and adverse-risk groups based on the presence of specific cytogenetic lesions and mutations [4]. *TP53* mutant (*TP53 Mut*) AML with >10% variant allele frequency (VAF) and myelodysplasia-related (*MR*) gene mutations are recognized as adverse risk. *MR* mutations include *ASXL1*, *BCOR*, and *EZH2*, which are key epigenetic genes, along with *RUNX1* and the genes that regulate the splicing process, such as *SF3B1*, *SRSF2*, *STAG2*, *U2AF1*, and *ZRSR2*. Adverse-risk cytogenetic abnormalities include t(v;11q23.3)/*KMT2A*-rearranged, t(8;16)(p11.2;p13.3)/*KAT6A::CREBBP*, t(6;9)(p23.3;q34.1)/*DEK::NUP214*, t(3q26.2;v)/*MECOM(EVI1)-*rearranged, inv(3)(q21.3q26.2) or t(3;3)(q21.3;q26.2)/*GATA2;MECOM(EVI1),* monosomy 5 or del(5q), monosomy 7, monosomy 17 or abnormal 17p, complex karyotype (CK), and monosomal karyotype (MK). While some recent validation studies have described the ELN 2022 as a precise prognostic tool that accurately stratifies survival among patients [5,6,7], others have recommended modifications as part of a continuous process to improve its effectiveness [8,9,10,11,12]. In particular, several modifications had been suggested in the ELN 2022 adverse-risk group.

Min-Yen Lo et al. proposed reclassifying AML with t(7;11)(p15;p15)/*NUP98::HOXA9* and AML with co-mutated *DNMT3A* and *FLT3-ITD* (without other favorable or adverse classifiers) as adverse because of their poorer prognostication [6]. It has also been suggested that *MR* mutations confer significantly better OS compared to other adverse-risk genotypes in patients who receive intensive induction therapy [11,13]. This finding has, however, been contradicted by other studies that identified similarly inferior survival rates in patients with other adverse genotypes, thus supporting the grouping of them together as adverse risk [14,15,16,17]. There have also been proposals to introduce a fourth ‘very-adverse-risk group’ that include *TP53 Mut* AML and selected cytogenetic aberrations based on the observed clinical outcomes that are distinctly worse when compared to other genotypes in the ELN 2022 adverse-risk group. *TP53 Mut* AML with a CK has been described as a genotype with extremely poor outcomes [18,19,20], suggesting that this group of patients should be assigned to a distinct “very-adverse-risk" category group [11,17]. Separately, AML with CK, MK, inv(3)(q21.3q26.2) or t(3;3)(q21.3;q26.2)/*GATA2*, *MECOM*(*EVI1*), or *TP53 Mut* have been proposed into this category based on their significantly worse complete remission (CR) rates, event-free survival (EFS), and OS [6]. It has also been suggested that AML patients with *TP53 Mut*, CK + *TP53 Mut*, or inv(3) could be grouped in an independent-risk category with a very poor prognosis [8,21]. These studies present diverse findings and suggestions regarding the genotype to be integrated into this very-adverse-risk category. Given its well-documented worse clinical outcomes and unique biological characteristics [22,23,24,25,26,27,28], *TP53 Mut* AML is now regarded as a distinct diagnostic entity separate from other subtypes of AML [4]. Its frequent co-occurrence with adverse cytogenetic lesions, such as CK, MK, and aneuploidy, including del(5q), −5, and −7, contributes to its poor prognosis [23,26], alongside a low incidence of co-mutation with non-adverse mutations like *FLT3, NPM1*, and *RAS* gene mutations when compared to *TP53* wild-type (*TP53-WT*) AML [29]. These unique clinical and biological profiles make it a potential independent marker of ultra-adverse risk, which is yet to be determined.

Although varied and at times conflicting, the shortcomings in the ELN 2022 adverse-risk group identified by these studies highlight the need to refine the ELN 2022 to more accurately cluster adverse-risk AML patients into a homogeneous, distinct, and reproducible risk group. Improving this tool will allow clinicians and scientists to make more precise predictions and comparisons on expected clinical outcomes and select the most appropriate and promising treatment strategies. Most recent studies conducted to validate the ELN 2022 were also limited in their patient selection, as some included patients on non-intense chemotherapy regimens, distinctly younger patient populations than the median age of 68 years, and very low numbers of AML *TP53* mutant patients. With these inadequacies, the findings from these studies may not precisely reflect the accuracy of the ELN 2022 in real-life practice.

Our study aims to illustrate the heterogeneous nature of the current ELN 2022 adverse-risk group, which limits its ability to accurately categorize non-M3 AML patients based on their OS in a real-world cohort. The study also suggests a refinement to classify these patients more precisely. Furthermore, we use a validation cohort to support our proposal.

## 2. Materials and Methods

### 2.1. Patients and Samples

We conducted a single-center retrospective cohort study at our institution involving non-APL AML patients diagnosed between 2016 and 2022. Patients with complete clinical and laboratory information, including cytogenetic and molecular testing data, were selected using the institution’s database. Furthermore, we used clinical and laboratory data from an external dataset [30] to validate our findings. AML diagnosis was reviewed and reclassified according to the 2022 International Consensus Classification (ICC) and further categorized into ELN 2022 risk groups using the proposed workflow by Lachowiez et al. [5]. We obtained study approval from the institution’s Research Ethics Board. Sample collection and the study procedure, including sample collection, were carried out in accordance with the principles outlined in the Declaration of Helsinki. Laboratory diagnostic procedures and evaluation, including bone marrow (BM) aspiration, cytogenetic testing, and molecular analyses, were all carried out in accordance with established guidelines. Clinical data, including age at diagnosis, gender, administered chemotherapy, allogeneic hematopoietic stem cell transplantation (allo-HCT) status, conventional karyotype analysis, next-generation sequencing (NGS) results, and survival status, were all collected. Those who did not receive intensive chemotherapy and those with incomplete clinical and laboratory data were excluded from the study.

### 2.2. Treatment

The study only included patients deemed fit for intensive chemotherapy (IC). These therapies consisted of 7 + 3 (cytarabine plus daunorubicin) and, separately, 7 + 3 plus midostaurin for *FLT3*-mutated AML. Other IC regimens included fludarabine, cytarabine, filgrastim, and idarubicin (FLAG-IDA), or a combination of mitoxantrone, etoposide, and modified high-dose cytarabine (Nove-HiDAC). Allo-HCT was administered to patients with intermediate- and high-risk AML at their first complete remission (CR). This was also considered for those with relapsed or refractory disease who achieved remission after salvage chemotherapy.

### 2.3. Cytogenetic Analysis

BM samples were analyzed using standard chromosome banding techniques and reported in accordance with the International System for Human Cytogenomic Nomenclature (ISCN) [31]. At least 20 metaphases were evaluated for each patient. Complex karyotype (CK) and monosomal karyotype (MK) were determined according to the European LeukemiaNet recommendations [4].

### 2.4. Molecular Analysis

Peripheral blood and BM samples were used for this analysis, from which total cellular RNA was extracted. *NPM1* mutation and *FLT3-ITD* status were evaluated using multiplex reverse transcriptase–polymerase chain reaction (PCR). In patients diagnosed before 2018, we conducted targeted sequencing (TAR-SEQ) using a 54-gene NGS myeloid panel [32], whereas a custom hybrid-capture-based 49 gene myeloid panel was used in those diagnosed after 2018 [33]. The detection limit for variant calling was established at 2%. Variants were subsequently annotated and classified as either pathogenic mutations or variants of unknown significance. We excluded variants classified as having unknown significance from the analysis. 

### 2.5. Statistical Analysis

Categorical variables were represented by numbers and percentages, while continuous variables were summarized by their median value and range. The relationships among variables were evaluated using the Chi-square test and Fisher’s exact test for categorical data. The Mann–Whitney U test and the Kruskal–Wallis test were applied in the assessment of continuous data variables. We determined OS by recording the duration in months from diagnosis to death from any cause or until the most recent follow-up, and analyzed it using the Kaplan–Meier method. Differences in OS among the risk groups were established using the log-rank test. The *p*-value, hazard ratio (HR), and 95% confidence interval (CI) were reported for each comparison. Moreover, *p*-values of less than 0.05 were considered statistically significant. Statistical analyses were conducted using GraphPad Prism software, version 10.3.1, as well as the IBM Statistical Package for the Social Sciences (SPSS), version 26. 

## 3. Results

### 3.1. Patient Characteristics

We studied 256 AML patients (median age 64 years; range 19–87 years), 140 (54.7%) of whom were male. At diagnosis, they had a median white blood cell (WBC) count of 7.2 (0.3–221) × 10^9^ cells/L, hemoglobin 8.7 (3.7–14.8) g/dL, platelets 64.5 (5–598) × 10^9^ cells/L and 48% (10–97%) BM blast percentage. All of the patients received intensive induction chemotherapy, with 169 (66%) achieving complete remission (CR). Only 99 individuals (38.7%) underwent allo-HCT as part of their treatment (Table 1).

**Figure 1:**
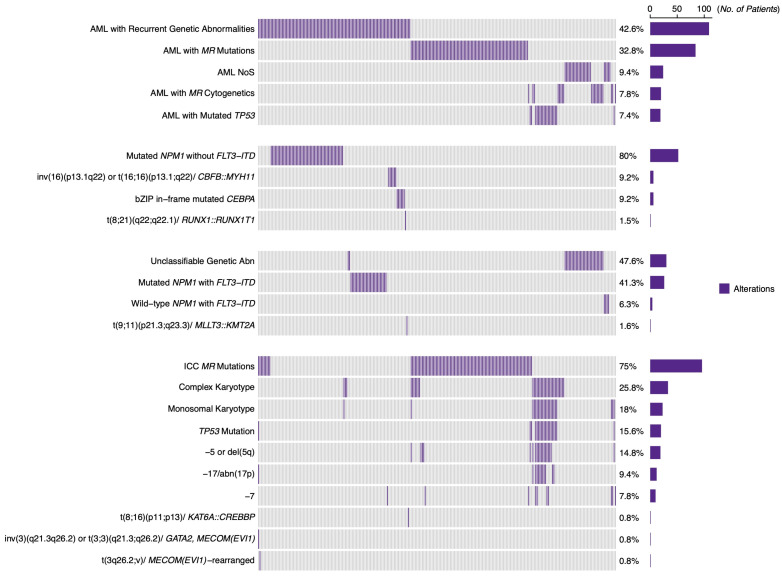
The 2022 ICC diagnostic classification and the ELN 2022 genetic profile of the study cohort. Note: Each column represents a patient and each row represents a genomic alteration. The right bar plot displays the percentage and number of patients with alterations. The first section of the heat map depicts genomic alterations according to the International Consensus Classification (ICC) of all patients in the study (n = 256), followed by alterations based on the ELN 2022 risk groups: favorable (n = 65), intermediate (n = 63), and adverse (n = 128). The alterations in each section are listed in descending order of frequency.

A total of 109 (42.6%) patients had AML with recurrent genetic abnormalities as defined by the 2022 ICC classification, whereas 84 (32.8%) and 19 (7.4%) were classified as having AML with *MR* mutations and AML with mutated *TP53*, respectively. Based on the ELN 2022 genetic risk classification, 65 (25.4%), 63 (24.6%), and 128 (50%) AML patients were categorized into favorable-, intermediate-, and adverse-risk groups, respectively (Figure 1). 

**Figure 2:**
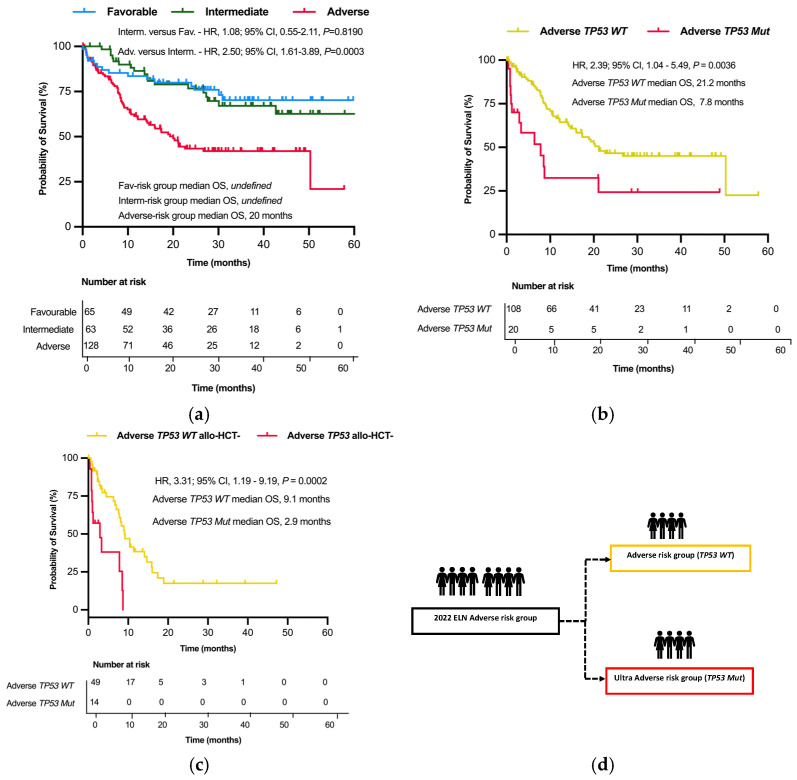
Kaplan–Meier survival plots of OS as per the ELN 2022 and the proposed refinement in the study cohort. (**a**) Kaplan–Meier plot of OS based on the ELN 2022 risk classification of all patients in the study. (**b**) Kaplan–Meier plot of OS among the ELN 2022 adverse *T53* mutant and *TP53* wild-type patients. (**c**) Kaplan–Meier plot of OS among the ELN 2022 adverse *T53* mutant and *TP53* wild-type patients in the study who did not receive allo-HCT (allo-HCT-). (**d**) Proposed refinement of the ELN 2022 adverse-risk group into adverse- (adverse *TP53 WT*) and ultra-adverse-risk (adverse *TP53 Mut*) groups.

There was no significant difference in CR rates among the three risk groups (*p* = 0.1819) after induction chemotherapy (Table 1). The Kaplan–Meier analysis based on the ELN 2022 risk classification showed shorter OS in the adverse-risk group (median OS, 20 months; *p* < 0.0001) than both the favorable- and intermediate-risk groups who did not attain the median survival. A pairwise comparison of the adverse- and intermediate-risk groups showed a significant difference in OS (*p* = 0.0003), but this was not significant between the intermediate- and favorable-risk groups (*p* = 0.819) (Figure 2a).

### 3.2. Impact of Allo-HCT on Overall Survival in the Study Cohort

**Figure A1:**
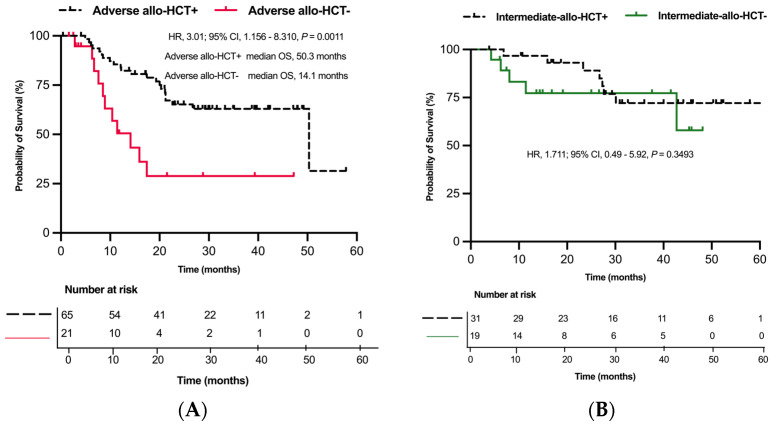

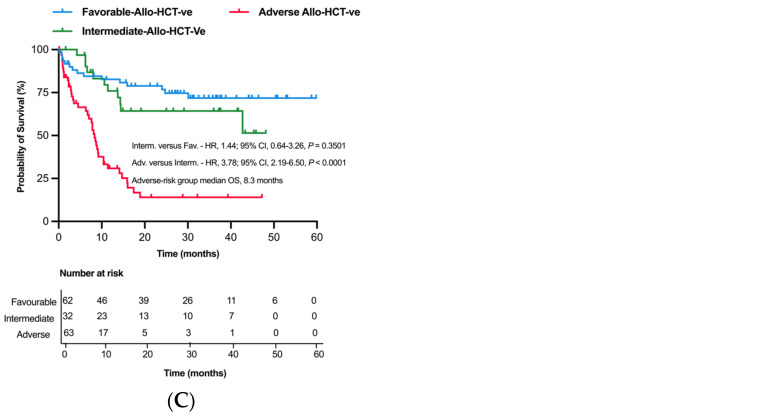
Kaplan–Meier survival plots showing the impact of allo-HCT on OS among the study participants as per the ELN 2022 risk groups. (**A**) OS between the ELN 2022 adverse-risk group who received allo-HCT (allo-HCT+) and those who did not (allo-HCT-). (**B**) OS between the ELN 2022 intermediate-risk group who received allo-HCT (allo-HCT+) and those who did not (allo-HCT-). (**C**) OS among all patients in the study who did not receive allo-HCT (allo-HCT-) categorized as per the ELN 2022.

A total of 31 (49.2%) and 65 (50.8%) patients in the intermediate- and adverse-risk categories, respectively, were the primary recipients of allo-HCT during their CR (Table 1). Only three (4.5%) patients in the favorable-risk category received allo-HCT. A total of 19 (14.8%) and 21 (33.3%) patients in the intermediate- and adverse-risk groups, respectively, achieved CR but were not transplanted. Allo-HCT recipients in the adverse-risk group had better OS than their counterparts who achieved CR but were not transplanted (median OS, 14.1 months versus 50.3 months; *p* = 0.0011) (Figure A1A). However, the difference in OS was not statistically significant (*p* = 0.3236) in the intermediate-risk group (Figure A1B). 

The Kaplan–Meier analysis of OS, following the removal (censoring) of allo-HCT recipients, indicated a significantly shorter OS in the adverse-risk group compared to the intermediate-risk group (median OS, 8.3 months versus *undefined*; *p* < 0.0001). However, no significant difference in OS was observed between the intermediate- and favorable-risk groups (*p* = 0.3501), which had not yet reached median survival (Figure A1C).

### 3.3. Heterogeneity and Proposed Refinement of the ELN 2022 Adverse-Risk Group 

The ELN 2022 adverse-risk group showed a higher prevalence of males (*p* = 0.0018), an older median age (*p* = 0.0151), low WBC count (*p* = 0.0023), low platelet count (*p* = 0.0284) and low BM blast percentage (*p* < 0.0001). (Table 1). *MR* mutations were the primary genetic abnormality reported among these patients (n = 96, 75%), followed by CK (n = 33, 25.8%) and MK (n = 23, 18%). Only 20 (15.6%) had *TP53 Mut* (Figure 1). Other adverse cytogenetics, like -5 or del(5q), −7, and -17, constituted 19 (14.8%), 10 (7.8%), and 11 (8.6%) patients in the adverse-risk group, respectively (Table 2). Moreover, t(8;16)(p11.2;p13.3)/*KAT6A::CREBBP* and inv(3)(q21.3q26.2) or t(3;3)(q21.3;q26.2)/*GATA2*, *MECOM (EVI1*) were each observed in only one participant, respectively (Figure 1).

We divided the ELN 2022 adverse-risk group into the following two subcategories based on the *TP53* mutation status: AML *TP53* mutant group (adverse *TP53 Mut*) and AML adverse *TP53* wild-type group (adverse *TP53 WT*). Notably, the adverse *TP53 Mut* group was characterized by a higher incidence of MK (<0.0001), CK (<0.0001), −5 or del(5q) (*p* < 0.0001), −7 (*p* = 0.0270), and −17 (*p* < 0.0001) cytogenetic lesions compared to the adverse *TP53 WT*. In addition, it showed a lower frequency of *MR* (*p* < 0.0001) and *FLT3-ITD* co-mutations (*p* = 0.0469) (Table 2).

Patients in the adverse *TP53 Mut* group had significantly shorter OS when compared to their adverse *TP53 WT* counterparts (median OS 7.8 months versus 21.2 months; *p* = 0.0036) (Figure 2b). This finding was also observed after excluding allo-HCT recipients from these subgroups (median OS 2.9 versus 9.1 months; *p* = 0.0002) (Figure 2c). Our proposed refinement of the ELN 2022 adverse-risk group categorizes it into the following two groups based on their differences in OS: adverse *TP53 Mut* and adverse *TP53 WT* groups. These two categories constitute our adverse- and ultra-adverse-risk groups, respectively (Figure 2d).

We separately analyzed the effect of the presence or absence of other ELN 2022 adverse-risk genotypes on OS in our study cohort. The presence of MK (*p* = 0.0002), CK (*p* < 0.0001), or −5 or del(5q) (*p* = 0.0042), −7 (*p* = 0.0474), and −17 (*p* < 0.0001) in AML patients from the ELN 2022 adverse-risk group was individually associated with an inferior OS. However, the presence or absence of specific *MR* mutations—*ASXL1* (*p* = 0.305), *BCOR* (*p* = 0.6617), *SRSF2* (*p* = 0.7178), *STAG2* (*p* = 0.6729), and *U2AF1* (*p* = 0.0924)—showed no significant difference in OS among these patients. Our analysis excluded *EZH2*, *SF3B1*, and *ZRSR2* alterations due to their low incidence (Table A1).

### 3.4. Validation of the Proposed ELN 2022 Refinement Using a Public Dataset

We examined 336 non-APL AML patients from the BEAT AML 2.0 study [30], with a median age of 58 years (range 18–79 years) who underwent intensive chemotherapy. Despite being a younger population, the biological sex distribution was similar to that of our cohort (Table A2). 

**Figure 3:**
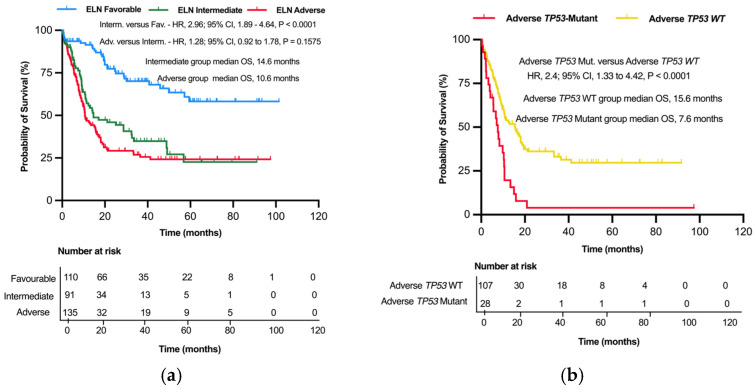
Kaplan–Meier survival plots of OS as per the ELN 2022 and proposed refinement in the validation cohort. (**a**) Kaplan–Meier plot of OS based on the ELN 2022 risk classification of the validation cohort. (**b**) Kaplan–Meier plot of OS among the ELN 2022 adverse *T53* mutant and *TP53* wild-type patients in the validation cohort.

A similar approach to that of our study population was used to first categorize and analyze the BEAT AML 2.0 study cohort according to the ELN 2022. A pairwise comparison of the ELN 2022 intermediate- and favorable-risk groups showed significant difference in OS (median OS, 14.6 months versus *undefined*; *p* < 0.0001). Even though the median OS was shorter in the adverse-risk group, this was not significantly different from the intermediate-risk group (median OS, 14.6 versus 10.6 months; *p* = 0.159) (Figure 3a).

We also subclassified the ELN 2022 adverse-risk group in the BEAT AML 2.0 cohort based on their *TP53* mutation status and analyzed the OS. The adverse *TP53 Mut* subgroup had significantly shorter OS when compared to the adverse *TP53 WT* group (median OS, 7.6 versus 15.6 months; *p* < 0.0001) (Figure 3b). 

## 4. Discussion

The widespread use of the ELN 2022 guideline is driven by the need for uniformity in definitions and standards for AML risk classification in clinical trials and practice using a relatively simple but accurate tool [4,11]. Studies continue to point out the limitations of this highly dynamic genetic risk stratification model with proposals to refine it for better performance in the real clinical world [6,8,9,11,13]. The ELN 2022 adverse-risk group comprises AML patients with diverse genetic profiles and biological characteristics, along with varying clinical outcomes [9,10,11]. We suggest refining the heterogeneous adverse-risk category based on the OS of non-APL AML patients undergoing intensive chemotherapy. 

We observed no significant difference in OS between the ELN 2022 intermediate- and favorable-risk groups (*p* = 0.8190) in our study cohort, which is in contrast with recent validation studies that found a distinct and effective separation between these two groups [5,6,9,11]. In accordance with current treatment standards, our study identifies higher allo-HCT rates in the ELN 2022 intermediate-risk group compared to the favorable-risk group [31 (49.2%) versus 3 (4.6%); *p* < 0.0001], which may have provided the intermediate-risk group with a survival advantage. The ELN 2022 adverse-risk group is heterogeneous, with its constituent genetic subtypes exhibiting widely varying clinical outcomes. *MR* mutations were the most frequently observed adverse-risk genotype in our study, comparable to previous studies, followed by CK and MK [6,7,9,10]. This group was also characterized by other adverse cytogenetics, like -5 or del(5q), −7, and −17. *TP53 Mut* AML, accounting for 20 (7.8%) patients, which is comparable to the reported incidence of 5–13% [27,28,29].

Our study identifies *TP53 Mut* AML as a distinct and homogeneous entity with poorer clinical outcomes within the ELN 2022 adverse-risk group. The low CR rate after induction chemotherapy, a short-lived remission status, and sub-optimal response to allo-HCT shortens the OS in these patients [26,28]. The adverse *TP53 Mut* AML patients in our study had the lowest CR rate (*p* = 0.0065) and an even worse OS (median OS 7.8 months; *p* = 0.0036), distinguishing them from the other adverse genotypes, referred to as adverse *TP53 WT* AML. Indeed, our median OS for *TP53 mut* AML patients aligns with other studies that reported a range from 1.8 months to 10 months [26]. *MR* mutations in AML have been reported as independent markers for adverse outcomes in de novo AML [14,17], but this is not as poor as the *TP53 Mut* AML, as observed in our study and previously [11,13]. Different studies have proposed that, alongside adverse *TP53 Mut* AML, other selected adverse-risk genotypes like AML with complex karyotype and inv(3)(q21.3q26.2) or t(3;3)(q21.3;q26.2)/*GATA2*, *MECOM(EVI1)* should be stratified as very adverse risk due to their significantly worse CR rates, EFS, and OS [5,6,26]. We were not able to analyze inv(3)(q21.3q26.2) or t(3;3)(q21.3;q26.2)/*GATA2, MECOM(EVI1*) in our study given their very small sample size (Figure 1). *TP53 Mut* AML has been found to frequently co-occur with adverse cytogenetic abnormalities, such as deletions of chromosomes 5 and 17, MK, and CK [23,26,28,29]. We observed a higher incidence of MK (*p* < 0.0001), CK (*p* < 0.0001), −5 or del(5q) (*p* < 0.0001), and −17 (*p* < 0.0001) among our adverse *TP53 Mut* patients, which has been attributed to ineffective DNA repair mechanisms and impaired apoptosis induced by *TP53* mutation in leukemic cells, indicating that *TP53* mutation could be the underlying genetic predisposition to these frequently co-occurring adverse cytogenetic lesions [23,29]. Consequently, it is not surprising that these adverse cytogenetics were associated with worse OS among our adverse-risk-group patients, considering their high frequency of co-occurrence with *TP53 Mut*. It has also been demonstrated previously that *TP53 Mut* AML is associated with a poor response to chemotherapy and reduced survival in patients, regardless of the presence of other aberration cytogenetics [29,34]. 

In our proposed refinement, we assign the ELN 2022 adverse *TP53 WT* and *TP53 Mut* AML into the adverse- and ultra-adverse-risk groups, respectively, given their distinctive OS. We believe it refines the ELN 2022 adverse-risk group more precisely in the setting of advancements in stem cell harvesting techniques, supportive care, and infection management. Since allo-HCT can obscure the adverse outcomes of patients in the adverse-risk group in our study, we ruled this out by observing comparable findings in allo-HCT-censored patients. Further, we validated the findings using a publicly available validation dataset from the Beat AML 2.0 cohort [30] that represents a robust, contemporary, and multicenter dataset.

Our study cohort comprised uniformly treated AML patients with intensive therapy (using cytarabine- and anthracycline-based induction regimens) with a broad age range (median age 63.5 years) that is closer to the median age of AML patients (68 years). Most of the studies conducted to validate the ELN 2022 comprised young patients, since most of those above 60 years do not qualify to receive intensive therapy. Our study’s findings could be constrained by its single-center design, limiting the generalizability to other contexts. Nevertheless, treatment protocols in this environment tend to be more consistent compared to multicenter studies. The few numbers of patients with high-risk cytogenetic abnormalities, such as inv (3)(q21.3q26.2) or t(3;3)(q21.3;q26.2)/*GATA2*, *MECOM*(*EVI1*), limited our subgroup analyses to determine their effect on OS in the adverse *TP53-WT* and *TP53 Mut* groups. Lastly, we used OS as the primary predicted outcome, which is comparable to other studies. However, it is known that patient mortality can result from treatment toxicity or other factors unrelated to the biology of the disease. Future multicenter studies with larger patient cohorts, especially comprising representative populations of all adverse genotypes, are necessary to confirm our findings and evaluate our proposed modification of the ELN 2022. Furthermore, it would be interesting to examine this refinement among patients treated with non-intensive induction chemotherapy.

## 5. Conclusions

In conclusion, the ELN 2022 adverse-risk group can be refined by categorizing patients receiving intensive chemotherapy into homogeneous risk groups that show distinct differences in OS under the current standard of care. We suggest separating this group into AML *TP53 Mut* and *TP53 WT* categories to create the “adverse-risk” and “ultra-adverse-risk” groups, respectively, among intensively treated patients.

## Figures and Tables

**Table 1:** Demographic and clinical characteristics of the study cohort.

Variables	All	Favorable	Intermediate	Adverse	*p*-Value
**Study Population, n (%)**	256 (100%)	65 (25.4%)	63 (24.6%)	128 (50%)	
**Age (y), median [range]**	64 [19–87]	63 [20–84]	60 [22–85]	65 [19–87]	0.0151 ^a^
**Male: Female Sex, n**	140:116	27:38	29:34	84:44	0.0018 ^b^
**WBC Count X 10^9^ cells/L, median [range]**	7.2 [0.3–221]	11 [0.6–197]	10.4 [0.4–221]	4.7 [0.3–137.6]	0.0023 ^a^
**Hemoglobin (g/dL), median [range]**	8.7 [3.7–14.8]	8.9 [6.2–13.3]	9.1 [5.3–14.1]	8.5 [11–14.8]	0.1368 ^a^
**Platelet X 10^9^ cells/L, n (%) median [range]**	64.5 [5–598]	88 [8–395]	72 [5–598]	53 [8–468]	0.0284 ^a^
**BM Blast %, median [range]**	48 [10–97]	64 [20–97]	51 [20–96]	39 [10–96]	<0.0001 ^a^
**Complete Remission, n (%)**	169 (66.0%)	42 (64.6%)	48 (76.2%)	79 (61.7%)	0.1819 ^b^
**Transplant, n (%)**	99 (38.7%)	3 (4.6%)	31 (49.2%)	65 (50.8%)	<0.0001 ^b^

Abbreviations: WBC, white blood cell; BM, bone marrow. ^a^—Kruskal–Wallis test. ^b^—Fisher’s exact test.

**Table 2:** Genotypic profile of adverse *TP53* mutant and *TP53* wild-type AML patients.

	ELN 2022 Adverse (n = 128)	Adverse *TP53* (n = 20)	Adverse *WT TP53* (n = 108)	*p-*Value
**Monosomal Karyotype ^¶^**	23 (18%)	17(85%)	6(5.6%)	<0.0001 *
**Complex Karyotype ^¶^**	33 (25.8%)	16 (80%)	17 (15.7%)	<0.0001 *
**−5 or del(5q) ^¶^**	19 (14.8%)	14 (70%)	5 (4.6%)	<0.0001 *
**−7 ^¶^**	10 (7.8%)	4 (20%)	6 (5.6%)	0.0270 *
**−17 ^¶^**	11 (8.6%)	10 (50%)	1 (0.9%)	<0.0001 *
***MR* Mutation †**	96 (75%)	3 (15%)	93 (86.1%)	<0.0001 *
***FLT3 -ITD*** **^ϕ^**	28 (21.9%)	1 (5%)	27 (25%)	0.0469 *
***NPM1*** **^ϕ^**	8 (6.2%)	0	8 (7.4%)	0.2087

NOTE. The ELN 2022 adverse-risk cytogenetics and mutations, along with other non-adverse-risk genotypes, are included in the table. **^¶^** Adverse-risk cytogenetics. † Adverse-risk mutations. ^ϕ^ Non-adverse-risk genotypes. * Statistically significant *p*-value.

## Data Availability

Data sharing statement: For original data, please contact hong.chang@uhn.ca.

## Abbreviations

The following abbreviations are used in this manuscript:

Allo-HCT	allogeneic hematopoietic stem cell transplantation
CIs	confidence intervals
CR	complete remission
EFS	event-free survival
FLAG-IDA	fludarabine, cytarabine, filgrastim, and idarubicin
HR	hazard ratio
ICs	intensive chemotherapies
ICC	International Consensus Classification
ISCN	International System for Human Cytogenomic Nomenclature
*MR*	myelodysplasia-related
NGS	next-generation sequencing
Nove-HiDAC	mitoxantrone, etoposide, and modified high-dose cytarabine
PCR	polymerase chain reaction
SPSS	Statistical Package for the Social Sciences
TAR-SEQ	targeted sequencing
*TP53 Mut*	*TP53* mutant
*TP53-WT*	*TP53* wild-type
OS	overall survival

## Appendix A

**Table A1:** Analysis of overall survival among AML patients based on each of the ELN 2022 adverse-risk genotypes.

Variable		ELN 2022 Adverse (n = 128)	Median OS (months)	HR	95% CI	*p-*Value
**MK**	*Present*	23 (18%)	7.8	2.822	1.27–6.26	0.0002
	*Absent*	105 (82%)	22.6			
**CK**	*Present*	33 (25.8%)	8.5	2.91	1.47–5.75	<0.0001
	*Absent*	95 (74.2%)	50.3			
**−5 or del(5q)**	*Present*	19 (14.8%)	7.8	2.0	0.86–4.63	0.0042
	*Absent*	109 (85.2%)	21.1			
**−7**	*Present*	10 (7.8%)	6.4	2.278	0.68–7.57	0.0474
	*Absent*	118 (92.2%)	20.3			
**−17**	*Present*	11 (8.6%)	3.1	5.18	1.45–18.48	<0.0001
	*Absent*	117 (91.4%)	21.2			
***RUNX1***	*Present*	43 (33.6%)	22.6	0.7642	0.46–1.27	0.305
	*Absent*	85 (66.4%)	18.9			
***ASXL1***	*Present*	37 (28.9%)	16.0	1.012	0.58–1.75	0.9667
	*Absent*	91 (71.1%)	20.0			
***SRSF2***	*Present*	36 (28.1%)	15.9	1.042	0.61–1.79	0.7178
	*Absent*	92 (71.9%)	20.3			
***U2AF1***	*Present*	21 (16.4%)	Undefined	0.5181	0.28–0.97	0.0924
	*Absent*	107 (83.6%)	18.9			
***STAG2***	*Present*	19 (14.8%)	15.9	1.21	0.59–2.49	0.6729
	*Absent*	109(85.2%)	20.3			
***BCOR***	*Present*	16 (12.5%)	10.4	1.169	0.53–2.57	0.6617
	*Absent*	112 (87.5%)	20.3			
***SF3B1***	*Present*	7 (5.5%)				
	*Absent*	121 (94.5%)				
***EZH2***	*Present*	2 (1.6%)				
	*Absent*	126 (98.4%)				
***ZRSR2***	*Present*	1 (0.8%)				
	*Absent*	127 (99.2%)				

NOTE. This table presents summarized findings from the Kaplan–Meier analysis of the overall survival (OS) for patients with each of the ELN 2022 adverse-risk genotypes, providing the median OS, hazard ratio (HR), 95% confidence interval (CI), and *p*-values. *EZH2*, *SF3B1*, and *ZRSR2* mutations are not analyzed due to their low incidence.

**Table A2:** Comparison of the baseline characteristics between the study and validation cohorts.

Variables		Study Population	Validation Cohort	*p*-Value
Age (years)	n	256	336	<0.0001 ^a^
	Median (min–max)	64 (19–87)	58 (18–79)	
Sex	Male	140 (54.7%)	182 (54.2%)	0.8997 ^b^
	Female	116 (45.3%)	154 (45.8%)	
ELN 2022	Favorable	65 (25.4%)	110 (32.7%)	0.0457 ^c^
	Intermediate	63 (24.6%)	91 (27.1%)	
	Adverse	128 (50%)	135 (40.2%)	
ELN Adverse-Risk Group	Adverse *TP53 WT*	108 (42.2%)	107 (31.8%)	0.283 ^b^
	Adverse *TP53 Mut*	20 (7.8%)	28 (8.3%)	
Transplant Status	Total Transplanted	99 (38.7%)	149 (44.3%)	0.1657 ^b^
	Allo-HCT	99 (38.7%)	137 (40.8%)	0.6048 ^b^
	DUCBT	None	12 (3.6%)	
	Favorable	3 (4.6%)	35 (31.8%)	<0.0001 ^c^
	Intermediate	31 (49.2%)	52 (57.1%)	
	Adverse	65 (50.8%)	62 (45.9%)	
	Adverse *TP53 WT*	59 (54.6%)	56 (52.3%)	0.9315 ^b^
	Adverse *TP53 Mut*	6 (30%)	6 (21.4%)	

DUCBT: Double-unit cord blood transplantation. ^a^—Mann–Whitney U test. ^b^—Chi-square test. ^c^—Fisher’s exact test.
